# Surgical biomicroscopy-guided intra-operative optical coherence tomography (iOCT) image super-resolution

**DOI:** 10.1007/s11548-022-02603-5

**Published:** 2022-04-01

**Authors:** Charalampos Komninos, Theodoros Pissas, Lina Mekki, Blanca Flores, Edward Bloch, Tom Vercauteren, Sébastien Ourselin, Lyndon Da Cruz, Christos Bergeles

**Affiliations:** 1 School of Biomedical Engineering & Imaging Sciences, King’s College London, London, SE1 7EU UK; 2 Wellcome/EPSRC Centre for Interventional and Surgical Sciences, University College London, London, W1W 7TS UK; 3 Moorfields Eye Hospital, London, EC1V 2PD UK; 4 Institute of Ophthalmology, University College London, London, EC1V 9EL UK

**Keywords:** iOCT, Image quality assessment, Super-resolution

## Abstract

**Purpose:**

Intra-retinal delivery of novel sight-restoring therapies will require the precision of robotic systems accompanied by excellent visualisation of retinal layers. Intra-operative Optical Coherence Tomography (iOCT) provides cross-sectional retinal images in real time but at the cost of image quality that is insufficient for intra-retinal therapy delivery.This paper proposes a super-resolution methodology that improves iOCT image quality leveraging spatiotemporal consistency of incoming iOCT video streams.

**Methods:**

To overcome the absence of ground truth high-resolution (HR) images, we first generate HR iOCT images by fusing spatially aligned iOCT video frames. Then, we automatically assess the quality of the HR images on key retinal layers using a deep semantic segmentation model. Finally, we use image-to-image translation models (Pix2Pix and CycleGAN) to enhance the quality of LR images via quality transfer from the estimated HR domain.

**Results:**

Our proposed methodology generates iOCT images of improved quality according to both full-reference and no-reference metrics. A qualitative study with expert clinicians also confirms the improvement in the delineation of pertinent layers and in the reduction of artefacts. Furthermore, our approach outperforms conventional denoising filters and the learning-based state-of-the-art.

**Conclusions:**

The results indicate that the learning-based methods using the estimated, through our pipeline, HR domain can be used to enhance the iOCT image quality. Therefore, the proposed method can computationally augment the capabilities of iOCT imaging helping this modality support the vitreoretinal surgical interventions of the future.

## Introduction

Regenerative therapies (e.g. [1, 2]) are emerging as treatments for blinding retinal diseases such as Age-Related Macular Degeneration [3]. Their efficiency, however, will depend on their precise injection in the sub-retinal and intra-retinal space. High-resolution cross-sectional (B-scans) images of the retina are required so that the retinal layers of interest can be visualised with quality adequate for injection guidance. Optical Coherence Tomography (OCT) captures such cross-sectional retinal images.

Intra-operative OCT (iOCT), acquired through recently introduced modified biomicroscopy systems such as Zeiss OPMI/Lumera and Leica Proveo/Enfocus, can be delivered in real time but at the expense of image quality (low signal strength and increased speckle noise [4]) with regard to pre-operative OCT. The produced iOCT scans are ambiguous and with limited interventional utility. While complementary research develops higher-quality iOCT systems, e.g. [5], we focus on computationally enhancing the capabilities of already deployed clinical systems.

An established approach to OCT quality enhancement is denoising. Spatially adaptive wavelets [6], Wiener filters [4], diffusion-based [7] and registration-based techniques [8] reduce speckle noise while preserving edges and image features. Unfortunately, these methods are limited by prolonged scanning periods, alignment errors and high computational cost, which limit their effectiveness for real-time interventions and iOCT.

Within the deep learning domain, Generative Adversarial Networks (GANs, [9]) can achieve image quality enhancement^1^ in natural images ([10–12]). Many of these approaches have been adopted for medical image quality enhancement [13] and cross-modality image synthesis [14]. Research has also been conducted for OCT denoising including [15–18], but these works do not focus on intra-operatively acquired OCT images.

Despite its superior quality, pre-operative OCT is acquired under different protocols (date, patient position, device) than iOCT, implying a domain gap in addition to deformations that may lead to generated images with artefacts. Therefore, our paper considers iOCT information only. We propose a methodology that uses high-resolution (HR) iOCT images generated offline through registered and fused low-resolution (LR) iOCT video frames (B-scans). Generated HR images are ranked for quality considering metrics that incorporate the quality of segmented retinal layers. High-scoring HR images comprise the target domain for image-to-image translation. Several image quality metrics and a complementary qualitative survey showcase that our super-resolution methodology improves iOCT image quality outperforming filter-based denoising methods and the learning-based state-of-the-art [19].

## Methods

This section presents the process of creating HR iOCT images, validating their quality and generating SR iOCT images through image-to-image translation.

### Data

**Fig. 1:**
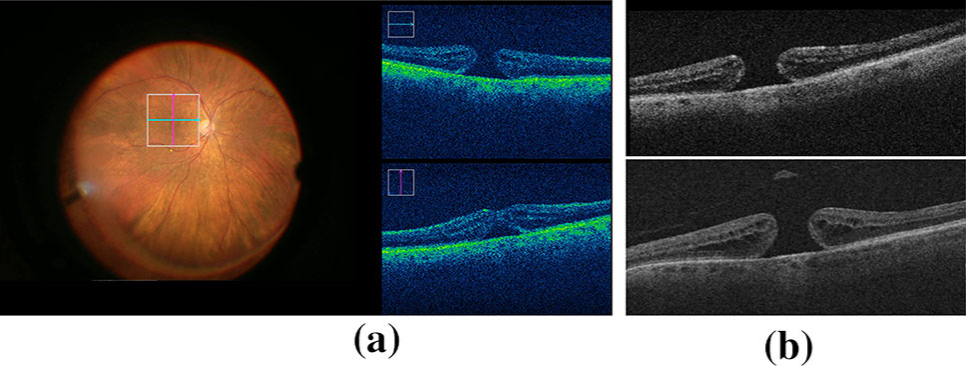
**a** Surgery video frame. Left: Biomicroscope view. Right: iOCT B-scans. **b** From top to bottom: intra-operative and pre-operative OCT images

Our data are derived from an internal Moorfields Eye Hospital database of vitreoretinal surgery videos, including intra-operative and pre-operative OCTs. We use a data-complete subset comprising 42 intra-operative retinal surgery videos acquired from 22 subjects. The data contain the surgical microscope view captured by a Zeiss OPMI LUMERA 700 with embedded LR iOCT frames (resolution of $440\hbox {x}300$) acquired by RESCAN 700 (see Fig. 1). These intra-operative sequences are used to generate HR iOCT images ($\widehat{HR}$), which are the target domain for the examined super-resolution models.

### $\widehat{HR}$ iOCT generation

**Fig. 2:**
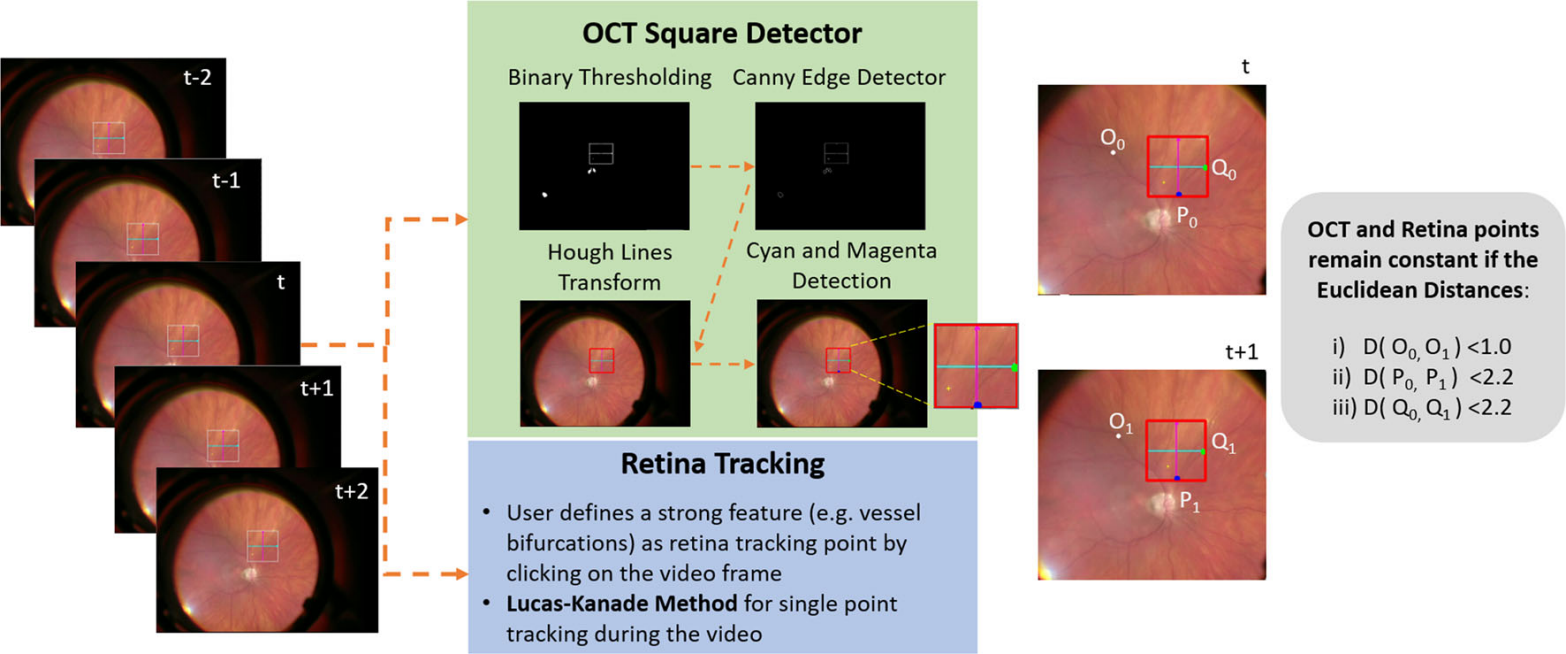
Overview of the proposed tracking methods for identifying the iOCT frames extracted from the same retina position

**Fig. 3:**
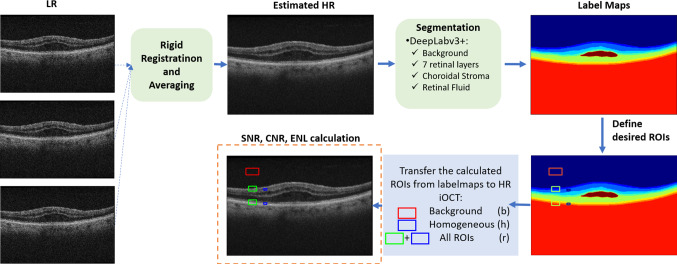
Fusion of multiple registered LR iOCT images through averaging and automatic extraction of ROIs for SNR, CNR and ENL calculation

Generating $\widehat{HR}$ iOCT images is based on fusing registered iOCT video frames that are acquired from the same retinal position by averaging the temporal information. This process is illustrated in Figs. 2 and 3.

First, for each surgery video (Fig. 1a), we identified the time intervals wherein both iOCT scan position and retina points positions remain constant. During such intervals, the acquired iOCT B-scans can be considered as corresponding to the same retinal location and can therefore be registered and fused to acquire a HR B-scan.

The position of the iOCT scan is obtained by detecting the white square depicted on the surgical microscope view (see Fig. 1a), which illustrates the iOCT’s scanning region. Detection starts with binary thresholding, Canny edge detection and Hough line transform on the microscope view image. To improve the robustness of identifying the iOCT scan position, we further detected the cyan and magenta arrows inside the already detected square (see Fig. 2). Two points (one point per arrow) were derived to represent the iOCT scan position.

Due to retina movement (patient breathing, surgical interactions) we must verify that the retina is also stationary. Therefore, we manually selected a point at the start of each video sequence that corresponds to a strong feature (e.g. vessel bifurcations), and tracked it using Lucas-Kanade method^2^.

If the aforementioned positions remained constant for more than eight consecutive video frames (number empirically selected), the corresponding iOCT B-scans were then rigidly registered to the first B-scan and averaged to generate the corresponding $\widehat{HR}$ iOCT frame (Fig. 3). We applied rigid registration as we wanted to avoid unrealistic deformations (e.g. folding) that non-rigid registration might introduce, damaging the quality and realism of the final averaged image. The fusion process led to a total of 1966 $\widehat{HR}$ images.

As the videos depict actual surgical procedures, many incoming LR iOCT images have low signal strength, calculated as signal to noise ratio (SNR) [20]. Thus, their corresponding fused $\widehat{HR}$ images will be of low SNR as well. Furthermore, imperfections in tracking retina points and registration errors between the LR iOCT images could lead to blurry averaged $\widehat{HR}$ iOCT scans. These factors affect the quality of many $\widehat{HR}$ images, which as a result lessens the robustness of the estimated $\widehat{HR}$ domain in terms of SNR and contrast.

To assess the quality of the generated images and define which ones should be included in the $\widehat{HR}$ dataset, we used three different metrics, i.e. SNR, Equivalent Number of Looks (ENL) and Contrast to Noise Ratio (CNR) [4]:

1 $$\begin{aligned} SNR= & {} 10log(max\{F_{lin}^2\}/\sigma _{lin}^2) \end{aligned}$$

2 $$\begin{aligned} ENL= & {} (1/H)\Sigma _{h=1}^{H}(\mu _{h}^{2}/\sigma _{h}^{2}) \end{aligned}$$

3 $$\begin{aligned} CNR= & {} (1/R)\Sigma _{r=1}^{R}(\mu _{r} - \mu _{b})/ \sqrt{\sigma _{r}^2+\sigma _{b}^2} \end{aligned}$$

where $F_{lin}$ is the linear magnitude image, $\sigma _{lin}$ the standard deviation of $F_{lin}$ in a background noise region, $\mu _{b}, \mu _{h}, \mu _{r},\sigma _{b}, \sigma _{h}, \sigma _{r}$ are the mean and standard deviations for background region (b), homogeneous regions (h) and all regions of interest (r), respectively. In our image quality assessment, we empirically used $H=2$ and $R=4$ (see Fig. 3). To obtain metrics describing image quality on key anatomical landmarks, namely, retinal layers, we compute retinal layer masks using a deep semantic segmentation model. Then, metric computation takes place for regions of interest (ROI) tightly cropped around retinal layers.

### Retinal layer segmentation

The segmentation model utilizes the architecture introduced in [21] and is trained using the Lovász-Softmax loss [22]. Due to the lack of large public pixel-level annotated datasets, we first pretrain the model for retinal fluid segmentation on the RETOUCH^3^ dataset, which contains 3200 images (72 subjects). The model was then fine-tuned for the task of retinal layer segmentation on the DUKE dataset^4^, which comprises 610 images (10 subjects). We qualitatively observed acceptable generalization of the segmentation model to our intra-operative OCT dataset. It is also worth mentioning that our aim is not a perfect segmentation of retinal layers but an acceptable approximation of the background area and pertinent retinal layers in the iOCT image in order to extract ROIs for the calculation of SNR, CNR and ENL.

Given the output label maps of the segmentation model, five ROIs are chosen (see Fig. 3): a background ROI (red rectangle), two small homogeneous ROIs on the second and the last retinal layers (blue rectangles), and two large ROIs on the first and the last retinal layers (green rectangles). The centre of the ROIs is random in the B-scan, so long as the aforementioned location constraints are respected, which stem from the requirements of the quality metrics themselves. Using (1–3), the ROIs, and considering empirically defined thresholds of 70.0, 3.0 and 10.0 for SNR, CNR and ENL, respectively, we identified 962 $\widehat{HR}$ images of acceptable quality to form the $\widehat{HR}$ dataset.

### Deep learning models

To perform super-resolution (SR), we used two state-of-the-art image-to-image translation models: CycleGAN [11] and Pix2Pix [12]. These models belong to the family of GANs which alternately train a generator *G* and a discriminator *D* in an adversarial manner. Pix2Pix requires supervision in the form of aligned image pairs to update its generator *G* as it minimizes the *L*1 loss between images of source (LR) and target ($\widehat{HR}$) domain. On the contrary, CycleGAN can be trained without the need of paired examples using cycle consistency to enforce mappings between forward ($G:LR\rightarrow \widehat{HR}$) and backward ($G:\widehat{HR}\rightarrow LR$) direction. Preliminary experiments, however, revealed that CycleGAN produced inconsistent results on unpaired images. We therefore also include *L*1 supervised losses for training CycleGAN.

### Implementation details

The dataset (962 image pairs of LR and $\widehat{HR}$ iOCT images) was split into three subsets: training set ($70\%$), validation set ($10\%$) and test set ($20\%$). We performed online data augmentation for the training set through rotation ($\pm 5^\circ$), translation($\pm 30$ width, $\pm 20$ height), horizontal flip (with a probability of 0.5), scale ($1\pm 0.2$) and the Albumentations^5^ ‘colorjitter’ augmentation with brightness and contrast between [2/3, 3/2]. Our implementations of Pix2Pix and CycleGAN are based on the code available online^6^, and both networks use CycleGAN’s ResNet-based generator [10] with 9 residual blocks. Our networks are trained using Adam Optimizer, for 200 epochs, with a batch size of 4 and input resolutions of $440\hbox {x}300$ for Pix2Pix and CycleGAN. Our experiments ran on an NVIDIA Quadro $\hbox {P}6000$ GPU with 24 GB memory.

## Results

This section presents the results of the quantitative and qualitative analysis that we performed to validate our SR pipeline. We also validate the merit of employing deep learning for this task by comparing our models with classical filter-based OCT denoising techniques and the learning-based state-of-the-art.

### Quantitative analysis

We quantitatively validate the quality enhancement of the SR images compared to the LR iOCT images. As our ground truth (HR) images are estimated by our methodology, full-reference metrics alone are not sufficient in image quality evaluation. Therefore, our analysis uses six different metrics including two full-reference metrics, i.e. Peak signal-to-noise ratio (PSNR) and Structural Similarity Index (SSIM) and four no-reference metrics, i.e. perceptual loss function (${\ell }_{feat}$) [10], Frechet Inception Distance (FID) [23], Global Contrast Factor (GCF) [24] and Natural Image Quality Evaluator (NIQE) [25]. The metric values were calculated on the test images of LR iOCT, SR using the state-of-the-art method of [19], SR using Pix2Pix [12] (SR-Pix) and SR using CycleGAN [11] (SR-Cyc). The evaluation metrics were computed on the original resolution (440x300px) for both Pix2Pix and CycleGAN outputs. The results are reported in Table 1. We assessed the statistical significance of the pairwise comparisons using paired t test. All p-values were $p < 0.001$ except for pairwise comparisons between SR-Cyc and filter-based methods for SSIM.

**Table 1:** Quantitative analysis. Arrows show whether higher/lower is better

	Full-Reference		No-Reference			
	PSNR $(\uparrow )$	SSIM $(\uparrow )$	${\ell }_{feat}(\downarrow )$	FID $(\downarrow )$	GCF $(\uparrow )$	NIQE $(\downarrow )$
LR	$21.99\pm 1.59$	$0.43\pm 0.12$	291.35	144.75	$\mathbf{7.52 }\pm \mathbf{0.76 }$	$\mathbf{7.10 }\pm \mathbf{1.48 }$
[19]	$22.11\pm 1.10$	$0.45\pm 0.08$	334.63	179.97	$7.14\pm 0.98$	$26.05\pm 2.16$
SR-Cyc	$\mathbf{25.83 }\pm \mathbf{1.85 }$	$\mathbf{0.58 }\pm \mathbf{0.10 }$	**120.99**	**56.64**	$5.9\pm 0.84$	$8.13\pm 1.07$
SR-Pix	$24.28\pm 1.65$	$0.48\pm 0.09$	171.24	70.12	$6.16\pm 0.80$	$12.76\pm 0.93$
Wiener	$24.49\pm 1.75$	$0.53\pm 0.10$	307.43	254.20	$5.49\pm 0.90$	$14.22\pm 3.64$
BM3D	$23.54\pm 1.76$	$0.54\pm 0.11$	348.12	187.10	$5.82\pm 0.95$	$14.99\pm 5.38$
SNN	$24.65\pm 1.81$	$0.56\pm 0.11$	275.43	171.99	$5.61\pm 0.88$	$14.62\pm 3.09$

Reference metrics (PSNR, SSIM) were calculated using $\widehat{HR}$ as reference images. As far as no-reference metrics are concerned, perceptual loss, ${\ell }_{feat}$, calculates the high-level perceptual similarity between two image domains by computing the distance of their feature representations extracted by Imagenet-pretrained Deep Convolutional Network [26]. We also used FID to capture how different are two image sets through the distance of their distributions of features extracted from the ImageNet-pretrained Inception-v3. Perceptual loss ${\ell }_{feat}$ and FID were calculated for the whole test dataset (193 images) of each image domain (LR, SR-Pix, etc.) with respect to the $\widehat{HR}$ domain. In addition, we trained a NIQE model on the test database of $\widehat{HR}$ images and assigned a NIQE score per test frame as well. The intuition behind the above three reference-free quality criteria is that if their values for SR images are lower than the corresponding values for LR, then our SR methodology generates images which are perceptually more similar to the $\widehat{HR}$ and thus of better quality. Finally, we used GCF, a no-reference metric which calculates the image contrast which is an essential characteristic for iOCT images.

**Fig. 4:**
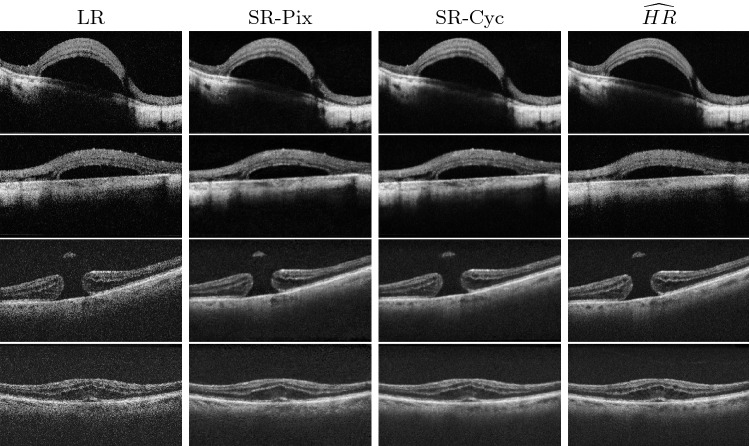
From left to right: LR, SR-Pix, SR-Cyc, $\widehat{HR}$

As shown in Table 1, SR-Cyc ranks first in terms of PSNR, SSIM ${\ell }_{feat}$ and FID, which shows that the image quality has been improved and is perceptually more similar to $\widehat{HR}$ (see also Fig. 4). Regarding GCF, the more noisy images (LR and SR output by [19]) exhibit higher values, probably due to the appearance of high-frequency information (speckle noise). Finally, for frames of size 440x300, SR-Cyc performs at 18.17 frames per second (FPS), while Pix2Pix at 17.51 which both are appropriate for iOCT real-time requirements.

### Qualitative analysis

To further validate our super-resolution pipeline, we performed qualitative analysis. Our survey included 20 pairs of LR and SR-Cyc images, randomly selected from the test set. We asked 8 retinal doctors/surgeons to evaluate these image pairs by assigning a score between 1 (strongly disagree) and 5 (strongly agree) on the following questions:

- **Q1**: Can you notice an improvement in the delineation of RPE/Bruchs vs. IS/OS junction in the generated image? (**A1**: 3.8±0.3)
- **Q2**: Can you notice a reduction of artefacts in the generated image?
  (**A2**: 3.9±0.1)
- **Q3**: Can you notice an improvement in the delineation of the ILM vs. RNFL in the generated image? (**A3**: 3.7±0.3)

Their answers, **A1**, **A2**, **A3** (mean±standard deviation), indicate that SR-Cyc images provide improved delineation of RPE vs IS/OS junction (Q1), reduction of artefacts (Q2) and improved delineation of ILM vs RNFL (Q3). Visual results are shown in Fig. 4, confirming the findings of our survey.

### Denoising results

To demonstrate the denoising effect of our work, as part of the broader aim of image quality enhancement, we compare our optimal (according to the metrics) network (SR-Cyc) with conventional denoising filters. We selected three different state-of-the-art speckle reduction methods for OCT images: Symmetric Nearest Neighbour (SNN) [27], adaptive Wiener filter [28] and BM3D [29] whose denoising ability has been assessed in several works [4, 18].

**Fig. 5:**
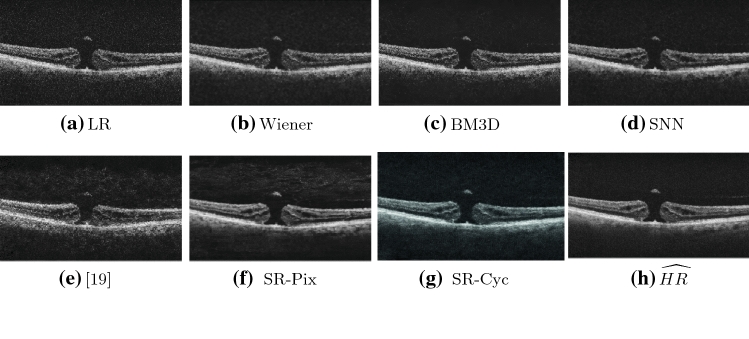
Visual results of different denoising methods

All the filter-based methods demonstrated considerable denoising capabilities, as shown in Fig 5. We can, however, observe that those filters blurred the images (b,c,d) and that retinal layers cannot be distinguished easily especially when compared to the outputs of SR-Pix and SR-Cyc. The SR-Cyc images, in particular, are visually more similar to the $\widehat{HR}$.

Quantitative analysis using the aforementioned metrics (see Table 1) shows that SR-Cyc achieved the best performance according to all metrics compared to the Wiener, BM3D and SNN filters. Among the filter-based techniques, SNN has the best performance according to PSNR, SSIM, ${\ell }_{feat}$, FID.

## Discussion and conclusions

This paper addresses the challenge of super-resolution in iOCT images. We overcome the absence of ground truth HR images by a novel pipeline that leverages spatiotemporal consistency of incoming iOCT B-scans to estimate the $\widehat{HR}$ images. Furthermore, we automatically assess the quality of the $\widehat{HR}$ images to accept only the high-scoring ones as target domain for super-resolution. Our quantitative and qualitative analysis demonstrated that the proposed super-resolution pipeline can achieve convincing results for iOCT image quality enhancement and outperform filter-based denoising methods with statistical significance. Future work will increase the sharpness of retinal layer delineations to produce iOCT images of quality even closer to pre-operative OCT scans.

## Footnotes

1 As is common in the literature, we interchange “quality enhancement” and “super-resolution”.

2 https://opencv.org/,calcOpticalFlowPyrLK.

3 https://doi.org/10.1109/TMI.2019.2901398.

4 https://doi.org/10.1364/BOE.6.001172.

5 https://albumentations.ai/.

6 https://github.com/junyanz/pytorch-CycleGAN-and-pix2pix.

## References

[1] Nazari H, Zhang L, Zhu D, Chader GJ, Falabella P, Stefanini F, Rowland T, Clegg DO, Kashani AH, Hinton DR, Humayun MS (2015). Stem cell based therapies for age-related macular degeneration: the promises and the challenges. Progress Ret Eye Res.

[2] da Cruz L, Fynes K, Georgiadis O, Kerby J, Luo YH, Ahmado A, Vernon A, Daniels JT, Nommiste B, Hasan SM, Gooljar SB, Carr AF, Vugler A, Ramsden CM, Bictash M, Fenster M, Steer J, Harbinson T, Wilbrey A, Tufail A, Feng G, Whitlock M, Robson AG, Holder GE, Sagoo MS, Loudon PT, Whiting P, Coffey PJ (2018). Phase 1 clinical study of an embryonic stem cell-derived retinal pigment epithelium patch in age-related macular degeneration. Nature Biotech.

[3] de Jong EK, Geerlings MJ, den Hollander AI (2020) Age-related macular degeneration. Genetics and genomics of eye disease, 155–180

[4] Ozcan A, Bilenca A, Desjardins AE, Bouma BE, Tearney GJ (2007). Speckle reduction in optical coherence tomography images using digital filtering. JOSA A.

[5] Viehland C, Keller B, Carrasco-Zevallos OM, Nankivil D, Shen L, Mangalesh S, Viet DT, Kuo AN, Toth CA, Izatt JA (2016). Enhanced volumetric visualization for real time 4D intraoperative ophthalmic swept-source OCT. Biomed Optic Exp.

[6] Adler DC, Ko TH, Fujimoto JG (2004). Speckle reduction in optical coherence tomography images by use of a spatially adaptive wavelet filter. Optic Lett.

[7] Bernardes R, Maduro C, Serranho P, Araújo A, Barbeiro S, Cunha-Vaz J (2010). Improved adaptive complex diffusion despeckling filter. Optics Exp.

[8] Sander B, Larsen M, Thrane L, Hougaard JL, Jørgensen TM (2005). Enhanced optical coherence tomography imaging by multiple scan averaging. Br J Ophthalm.

[9] Goodfellow I, Pouget-Abadie J, Mirza M, Xu B, Warde-Farley D, Ozair S, Courville A, Bengio Y (2014) Generative adversarial nets. Advances in neural information processing systems, 27

[10] Johnson J, Alahi A, Fei-Fei L (2016) Perceptual losses for real-time style transfer and super-resolution. In: European conference on computer vision, pp. 694–711 . Springer, Berlin

[11] Zhu J-Y, Park T, Isola P, Efros AA (2017) Unpaired image-to-image translation using cycle-consistent adversarial networks. In: Proceedings of the IEEE International conference on computer vision, pp. 2223–2232

[12] Isola P, Zhu J-Y, Zhou T, Efros AA (2017) Image-to-image translation with conditional adversarial networks. In: Proceedings of the IEEE conference on computer vision and pattern recognition, pp. 1125–1134

[13] Ravì D, Szczotka AB, Pereira SP, Vercauteren T (2019). Adversarial training with cycle consistency for unsupervised super-resolution in endomicroscopy. Med Image Anal.

[14] Wolterink JM, Dinkla AM, Savenije MH, Seevinck PR, van denBerg CA, Išgum I(2017) Deep mr to ct synthesis using unpaired data. In: International Workshop on Simulation and Synthesis in Medical Imaging, pp. 14–23 . Springer, Berlin

[15] Devalla SK, Subramanian G, Pham TH, Wang X, Perera S, Tun TA, Aung T, Schmetterer L, Thiéry AH, Girard MJ (2019). A deep learning approach to denoise optical coherence tomography images of the optic nerve head. Scientif Report.

[16] Apostolopoulos S, Salas J, Ordóñez JL, Tan SS, Ciller C, Ebneter A, Zinkernagel M, Sznitman R, Wolf S, De Zanet S, Munk MR (2020) Automatically enhanced oct scans of the retina: a proof of concept study. Scientif Report 10(1):1–8 10.1038/s41598-020-64724-8 PMC7210925 32385371

[17] Lazaridis G, Lorenzi M, Ourselin S, Garway-Heath D (2021). Improving statistical power of glaucoma clinical trials using an ensemble of cyclical generative adversarial networks. Med Image Anal.

[18] Halupka KJ, Antony BJ, Lee MH, Lucy KA, Rai RS, Ishikawa H, Wollstein G, Schuman JS, Garnavi R (2018). Retinal optical coherence tomography image enhancement via deep learning. Biomed Optics Exp.

[19] Komninos C, Pissas T, Flores B, Bloch E, Vercauteren T, Ourselin S, Cruz LD, Bergeles C(2021) Intra-operative oct (ioct) image quality enhancement: a super-resolution approach using high quality ioct 3d scans. In: International workshop on ophthalmic medical image analysis, pp. 21–31 . Springer, Berlin

[20] Hardin JS, Taibbi G, Nelson SC, Chao D, Vizzeri G (2015). Factors affecting cirrus-hd oct optic disc scan quality: a review with case examples. J Ophthalmol.

[21] Chen L-C, Zhu Y, Papandreou G, Schroff F, Adam H, Ferrari V, Hebert M, Sminchisescu C, Weiss Y (2018). Encoder-decoder with atrous separable convolution for semantic image segmentation. Computer Vision - ECCV 2018.

[22] Berman M, Triki AR, Blaschko MB (2018) The lovász-softmax loss: A tractable surrogate for the optimization of the intersection-over-union measure in neural networks. In: Proceedings of the IEEE conference on computer vision and pattern recognition (CVPR)

[23] Heusel M, Ramsauer H, Unterthiner T, Nessler B, Hochreiter S(2017) Gans trained by a two time-scale update rule converge to a local nash equilibrium. Advances in neural information processing systems, 30

[24] Matkovic K, Neumann L, Neumann A, Psik T, Purgathofer W (2005). Global contrast factor-a new approach to image contrast. Computat Aesthet.

[25] Mittal A, Soundararajan R, Bovik AC (2012). Making a completely blind image quality analyzer. IEEE Signal Process Letter.

[26] Russakovsky O, Deng J, Su H, Krause J, Satheesh S, Ma S, Huang Z, Karpathy A, Khosla A, Bernstein M, Berg AC, Fei-Fei L (2015). Imagenet large scale visual recognition challenge. Int J Comput Vision.

[27] Harwood D, Subbarao M, Hakalahti H, Davis LS (1987). A new class of edge-preserving smoothing filters. Pattern Recognit Letter.

[28] Bakker P, van Vliet L.J, Verbeek PW (1999) Edge preserving orientation adaptive filtering. In: Proceedings. 1999 IEEE computer society conference on computer vision and pattern recognition (Cat. No PR00149), 1, pp. 535–540

[29] Dabov K, Foi A, Katkovnik V, Egiazarian K (2007). Image denoising by sparse 3-d transform-domain collaborative filtering. IEEE Transact Image Process.

